# Setting the Research Agenda on the Health Effects of Chemicals

**DOI:** 10.3390/ijerph110101049

**Published:** 2014-01-14

**Authors:** Keri Fulcher, Gibb Herman

**Affiliations:** Tetra Tech Sciences, 1320 North Courthouse Road, Suite 600, Arlington, VA 22201, USA; E-Mail: keri.fulcher@tetratech.com

**Keywords:** CICAD, EHC, WHO, chemicals, research, disease burden, UNEP

## Abstract

In 2011, World Health Organization (WHO) scientists reported that a significant percentage of global deaths and disability-adjusted life years (DALYs) in 2004 could be attributed to chemicals. The 2011 review focused only on certain chemicals, however, and concluded that the global burden of disease was underestimated because of serious data gaps. While various chemical assessment documents have identified research needs for individual chemicals, a systematic review of such documents to identify research themes that could be applied to the multitude of chemicals for which there is little information has not been done. Even for chemicals for which there are considerable data, the information is not sufficient to make an estimate of the chemical’s contribution to the burden of disease. The WHO Environmental Health Criteria (EHC) documents and Concise International Chemical Assessment Documents (CICADs) identify research needs or data gaps in our knowledge of chemicals. We identified several common themes in these documents and in documents prepared by WHO on 10 chemicals of major public health concern. These themes include biomarkers, longitudinal epidemiological studies, mechanisms of disease, reproductive and developmental effects and exposure assessment. Specific examples of data gaps culled from more than 300 WHO documents provide researchers with specific topics for further research.

## 1. Background

Prüss-Ustün *et al.* estimated that in 2004, 4.9 million deaths (8.3% of deaths worldwide) and 86 million disability-adjusted life years (5.7% of DALYs worldwide) could be attributed to selected chemicals [1]. The authors conclude, however, that even these estimates significantly underestimate the real burden of disease due to chemicals [1]. Two reasons cited by Prüss-Ustün *et al*. for the underestimation are (1) the lack of exposure–response relationships for many chemicals and (2) the lack of exposure data [1]. Even for chemicals with health consequences supported by strong evidence of causality, there are significant knowledge gaps. Chemicals not included in the Prüss-Ustün *et al*. analysis because of limited data include mercury, dioxins, organic chlorinated solvents, polychlorinated biphenyls (PCBs), and chronic pesticide exposures [2]. A 2013 report by the World Health Organization (WHO) and UNEP states that the disease risk from endocrine disrupting chemicals may be seriously underestimated [3]. In 2013, the United Nations Environment Program (UNEP) echoed the concern of Prüss-Ustun *et al*. that little is known about the global disease burden attributable to chemicals [2]. 

Of the tens of thousands of chemicals on the market, only a fraction has been thoroughly evaluated to determine their effects on human health and the environment [2]. These same chemicals have been evaluated over and over by various international, national, and nongovernmental organizations. Even for the chemicals for which we have a great deal of information, there are serious data gaps. A 1999 report by the European Chemicals Agency found that of 2,500 high-production-volume chemicals, only 14% had sufficient data to comply with the basic requirements in the European Union’s Dangerous Substances Directive, 65% had incomplete data, and 21% had no data at all [2]. A 1998 report by the USA Environmental Protection Agency found that of approximately 3,000 substances sold above one million pounds per year, only 7% had minimum data considered to be necessary by the Organization for Economic Cooperation and Development (OECD), and 43% had no data at all [2]. Despite some improvement, UNEP states that these figures continue to be indicative of the problem [2].

Research to address these gaps in our knowledge is presented in chemical assessment documents prepared by various international, national and nongovernmental organizations. Many of the research needs identified by these organizations for given chemicals are similar. Furthermore many of the research needs across chemicals are similar. The purpose of the current review is to identify common research themes. For our review, we utilized WHO chemical assessment documents. WHO relies on international experts for its assessments and is a recognized leader in chemical risk assessment. 

## 2. Methodology

The WHO documents included in this review are Environmental Health Criteria (EHC) documents [4], Concise International Chemical Assessment Documents (CICADs) [5], and WHO documents on “10 chemicals of major public health concern” [6]. The “Further Research” sections of the EHCs, the “Uncertainties” sections of the CICADs and the WHO documents on “10 Chemicals of Major Public Health Concern” were examined. All documents on the 10 Chemicals of Major Public Health Concern and on methodologies were considered regardless of publication date. For other chemicals, documents published between 2002 and 2012 were reviewed. Each of these documents was examined to determine: (1) the research needs specific to the chemical, and (2) the commonality of these needs to the research needs expressed in other WHO chemical assessment documents. Each research need identified in the WHO documents was accepted at face value. No attempt was made to question the research need stated by the international panel of experts. Five common themes of research needs were identified; specific examples of these needs are provided. 

## 3. Five Major Research Themes

### 3.1. Research on Biomarkers

Biomarkers are categorized as markers of exposure, susceptibility and effect [7]. Selection of biomarkers provides the opportunity for greater precision in risk assessment [8]. For several chemicals, including fluoride [9] and nitrobenzene [10], robust exposure biomarkers need to be identified. For other chemicals, biomarkers have been identified, but further study is needed. For example, the EHC on nitropolycyclic aromatic hydrocarbons describes a need to assess the sensitivity of the exposure biomarkers [11]. The cadmium EHC suggests further study on beta-2-microglobulin as a biomarker of exposure and effect [12]. Monitoring of biomarkers of exposure could allow for earlier intervention, thus reducing the incidence of adverse health effects [8], but validation of such biomarkers is needed (e.g., validation of urinary muconic acid and macromolecular adducts as biomarkers of benzene exposure [13]).

### 3.2. Longitudinal Epidemiological Studies

Longitudinal studies are essential to understanding the long-term effects of exposure [14]. The EHC on arsenic describes the need for longitudinal studies on cardiovascular morbidity, hypertension, diabetes, neurological effects and reproductive endpoints [15]. Cadmium-exposed individuals demonstrating beta-2-microglobulinuria should be observed longitudinally to establish the nature, severity and prognosis of health outcomes [12]. Case reports, case series and cross-sectional studies suggest that occupational exposures to bentonite, kaolin and selected clay minerals are associated with respiratory effects, and longitudinal studies of such occupational exposures are needed [16]. Case reports and cross-sectional studies suggest that palladium is a sensitizing agent, and longitudinal studies of the incidence of allergic (and other) diseases in palladium-exposed workers are recommended [17]. Further epidemiological research on hematological cancer, blood changes and genotoxic effects at both low and high exposure levels is a high priority for benzene [13]. Cancer epidemiological studies of dichlorodiphenyltrichloroethane (DDT) are needed to address current inconsistencies in results [18]. There is a significant lack of epidemiological data on nitrobenzene [10], nitropolycyclic aromatic hydrocarbons [11], butyl acetates [19] and asphalt (bitumen) [20]. 

### 3.3. Understanding Mechanisms of Disease

Understanding the mechanisms of disease provides insight into how chemicals cause disease and how disease may be prevented or treated. Research on mechanisms sheds light on the relationship between external exposure and internal dose [8]. Several EHCs and CICADs noted a need for basic research on the mechanisms of carcinogenesis [12,13,15,21]. Further research is needed on the mechanisms associated with fluoride’s clastogenicity [9] and the accumulation and mobilization of inorganic lead from bone [22]. There is considerable uncertainty regarding the mechanisms of carcinogenicity with respect to bromoethane [23] and formaldehyde [24]. Research examining mechanisms of carcinogenesis will assist in the identification of appropriate biomarkers [8]. 

### 3.4. Research on Reproductive and Developmental Effects

Reproductive and developmental health issues have gained public attention because of concern over endocrine disruptors, thus reinforcing anxiety over the role of environmental agents [25,26]. Preconception and *in utero* exposures have been linked to reproductive effects, including fetal loss, malformation, low birth weight, infertility and subfertility [25]. Heavy metals, including lead, cadmium and mercury, have been found in breast milk and are known to be associated with a range of adverse health effects [25]. Further research is needed on the effects of cadmium on the placenta and fetus [12]. The EHC on lead describes the need to assess the effects of prenatal and postnatal exposure to lead and to improve the definition of paternally mediated lead exposure [22]. Additional studies of the effects of fluoride [9], palladium [17] and ethylene oxide [21] on reproductive outcomes are critically needed. There is a complete lack of data on reproductive effects for many chemicals, including nitrobenzene [10], 4-chloroaniline [27], coal tar creosote [28], cyclic acid anhydrides [29] and strontium and strontium compounds [30]. 

### 3.5. Exposure Assessment

The goal of exposure assessment is to gather data on the route, magnitude, duration, frequency and distribution of exposures to hazardous agents for individuals and populations [31]. The EHC on cadmium expresses a need to assess human exposure from all types of media through environmental monitoring [12]. Additional exposure information is needed for 2-methoxyethanol [32], 2-butoxyethanol [32] and ethylene glycol [33] from consumer products, for diethylene glycol dimethyl ether [34] in cosmetics and for diethyl phthalate [35] from medical devices. Some chemicals lack data on exposure in particular settings (e.g., occupational, residential). For example, occupational exposure data are needed for bromoethane [23], palladium [17], 4-chloroaniline [27], glyoxal [36] and thiourea [37], whereas environmental exposure data are required for nitrobenzene [10], palladium [17], 2-butoxyethanol [32] and butyl acetates [19]. The EHC on DDT describes the need for studies regarding occupational and residential exposures and infant exposure via breast milk [38]. Other chemicals lack data on exposure via specific routes (e.g., 1,2,3-trichloropropane [39] through dermal exposure; ethylene glycol [33] and DDT [38] by ingestion). Little, if any, information on exposure is available for some chemicals (e.g., nitropolycyclic aromatic hydrocarbons [11], arsine [40], coal tar creosote [28], strontium and strontium compounds [30], brominated phenols [41]). 

## 4. Discussion

Understanding the effects of chemicals at environmental levels of exposure underlies the research themes described above. Most of the chemical assessment documents included in this review were produced in the past decade. Even for the older documents, however, the research themes are similar. 

Prüss-Ustün *et al.* stated that one reason for their underestimation of the burden of disease from chemicals was that their analysis failed to capture health impacts from exposure to polluted sites, which are estimated to put more than 56 million people at risk worldwide [1]. Even 56 million may be an underestimate. E-waste, for example, is growing dramatically. E-waste describes end-of-life electrically powered devices such as computers, television sets, and cell phones. E-waste contains a wide range of inorganic contaminants such as lead, cadmium, and mercury and organic pollutants such as PCBs, brominated flame retardants, and phthalates [42]. In 2005, the global volume of e-waste was estimated at 20–50 million tonnes and growing at a rate of 3%–5% per year [42]. The major producers of e-waste are the United States, the European Union, China, and Japan, but the contribution of developing countries is expected to increase dramatically in the next decade [42]. In South Africa and China, UNEP predicts that by 2020, e-waste from old computers is expected to jump 200%–400% from 2007 levels [43]. In India, it is expected to jump 500%. In China the e-waste in 2020 from mobile phones is expected to increase by seven times from 2007 levels. In India, it is expected to increase 18 times [43]. 

Much of the available health information on chemicals is the result of studies of acute response (e.g., poisonings, allergic response, *etc.*). Less is known about subtle long-term effects, such as kidney or liver disease. By 2030, chronic diseases are projected to cause nearly five times as many deaths as infectious diseases worldwide [2]. 

The shift in the global burden of disease from communicable to noncommunicable disease (NCD) over the period 1990 to 2010 has been dramatic [44]. NCDs are now the principal cause of sickness and death throughout the world [45], and exposure to chemicals contribute to a wide range of NCD [2]. Fifty-four percent of the global burden of disease attributed to chemicals is borne by children under the age of 15 [1]. 

Over the period 2012 to 2020, chemical production is expected to increase approximately 25% in North America and Europe, 40% in Africa and the Middle East, and 46% in the Asia-Pacific region [2]. The most rapid growth is expected in India and China (59% and 66%, respectively) [2]. This increase in chemical production, particularly in developing countries, can be expected to occur without chemical management procedures [2]. Continued growth of the chemical industry especially in lower- and middle-income countries increases the risk of chemical exposure and ill-health [46]. A better understanding of chemical risks, however, will improve chemical risk management procedures [46]. 

A 2013 report by UNEP and WHO states that there is a growing probability that maternal, fetal and childhood exposure to chemical pollutants play a larger role in the etiology of many endocrine diseases and disorders of the thyroid, immune, digestive, cardiovascular, reproductive and metabolic systems (including childhood obesity and diabetes) than previously thought possible [3]. Despite the attention given to endocrine disrupting chemicals (EDCs) over the last decade, the 2013 report on EDCs describes a critical need for longitudinal epidemiology studies [3], one of the themes identified in our review. 

Another of the research themes identified in this review is biomarkers. In 2009, the International Conference on Chemicals Management (ICCM) (comprising governments and other stakeholders) included biomonitoring as an indicator for tracking the effectiveness of sound chemical management programs [47].Despite this emphasis by ICCM, biomonitoring programs are confined to developed countries, and in most of those countries, the programs evaluate only a relatively small number of pollutants [2]. Thus, even in a research area where an international body has recommended greater effort, there continues to be a paucity of information.

## 5. Conclusion

As indicated by Prüss-Ustün *et al.*, the global burden of disease due to chemical exposure is considerable, but it is an underestimate [1]. To better understand the magnitude of the burden requires additional research in the five theme areas that we have identified. Although research needs have been identified for various chemicals, this is the first systematic review of chemical assessment documents to identify common research needs. Until such data gaps are filled, the global burden of disease due to chemicals will continue to be seriously underestimated.

## References

[1] Prüss-Ustün A., Vickers C., Haefliger P., Bertollini R. (2011). Knowns and unknowns on burden of disease due to chemicals: A systematic review. Environ. Health.

[2] (2013). Global Chemicals Outlook.

[3] (2013). State of the Science of Endocrine Disrupting Chemicals—2012.

[4] International Programme on Chemical Safety: Environmental Health Criteria. http://www.who.int/ipcs/publications/ehc/en/.

[5] International Programme on Chemical Safety: Concise International Chemical Assessment Documents. http://www.who.int/ipcs/publications/cicad/en/.

[6] International Programme on Chemical Safety: Ten chemicals of Major Public Health Concern. http://www.who.int/ipcs/assessment/public_health/chemicals_phc/en/.

[7] (2001). Environmental Health Criteria 222—Biomarkers in Risk Assessment: Validity and Validation.

[8] (1993). Environmental Health Criteria 155—Biomarkers and Risk Assessment: Concepts and Principles.

[9] (2002). Environmental Health Criteria 227—Fluorides.

[10] (2003). Environmental Health Criteria 230—Nitrobenzene.

[11] (2003). Environmental Health Criteria 229—Selected Nitro- and Nitro-Oxy-Polycyclic Aromatic Hydrocarbons.

[12] (1992). Environmental Health Criteria 134—Cadmium.

[13] (1993). Environmental Health Criteria 150—Benzene.

[14] (1999). Environmental Health Criteria 210—Principles for the Assessment of Risks to Human Health from Exposure to Chemicals.

[15] (2001). Environmental Health Criteria 224—Arsenic and Arsenic Compounds.

[16] (2005). Environmental Health Criteria 231—Bentonite, Kaolin, and Selected Clay Minerals.

[17] (2002). Environmental Health Criteria 226—Palladium.

[18] (1979). Environmental Health Criteria 9—DDT and its Derivatives.

[19] (2005). Concise International Chemical Assessment Document 64—Butyl Acetates.

[20] (2004). Concise International Chemical Assessment Document 59—Asphalt (Bitumen).

[21] (2003). Concise International Chemical Assessment Document 54—Ethylene Oxide.

[22] (1995). Environmental Health Criteria 165—Inorganic Lead.

[23] (2002). Concise International Chemical Assessment Document 42—Bromoethane.

[24] (2002). Concise International Chemical Assessment Document 40—Formaldehyde.

[25] (2001). Environmental Health Criteria 225—Principles for Evaluating Health Risks to Reproduction Associated with Exposure to Chemicals.

[26] (1984). Environmental Health Criteria 30—Principles for Evaluating Health Risks to Progeny Associated with Exposure to Chemicals During Pregnancy.

[27] (2003). Concise International Chemical Assessment Document 48—4-Chloroaniline.

[28] (2004). Concise International Chemical Assessment Document 62—Coal Tar Creosote.

[29] (2009). Concise International Chemical Assessment Document 75—Cyclic Acid Anhydrides: Human Health Aspects.

[30] (2010). Concise International Chemical Assessment Document 77—Strontium and Strontium Compounds.

[31] (2000). Environmental Health Criteria 214—Human Exposure Assessment.

[32] (2010). Concise International Chemical Assessment Document 67—Selected 2-Alkoxyethanols.

[33] (2002). Concise International Chemical Assessment Document 45—Ethylene Glycol: Human Health Aspects.

[34] (2002). Concise International Chemical Assessment Document 41—Diethylene Glycol Dimethyl. Ether.

[35] (2003). Concise International Chemical Assessment Document 52—Diethyl Phthalate.

[36] (2004). Concise International Chemical Assessment Document 57—Glyoxal.

[37] (2003). Concise International Chemical Assessment Document 49—Thiourea.

[38] (2011). Environmental Health Criteria 241—DDT in Indoor Residual Spraying: Human Health Aspects.

[39] (2003). Concise International Chemical Assessment Document 56—1,2,3-Trichloropropane.

[40] (2002). Concise International Chemical Assessment Document 66—2,4,6-Tribromophenol and Other Simple Brominated. Phenols.

[41] (2005). Concise International Chemical Assessment Document 66—2,4,6-Tribromophenol and Other Simple Brominated. Phenols.

[42] (2005). E-waste, the Hidden Side of IT Equipment’s Manufacturing and Use.

[43] (2009). Recycling—From E-waste to Resources.

[44] Murray C., Vos T., Lozano R., Naghavi M., Flaxman A.D., Michaud C., Ezzati M., Shibuya K., Salomon J.A., Abdalla S. (2012). Disability-adjusted life years (DALYs) for 291 diseases and injuries in 21 regions, 1990–2010: A systematic analysis for the global burden of disease study 2010. Lancet.

[45] (2011). Global Status Report on Noncommunicable Diseases 2010.

[46] (2011). WHO Public Health and Environment Global Strategy Overview 2011.

[47] International Conference on Chemicals Management Strategic Approach to International Chemicals Management. Proceedings of the International Conference on Chemicals Management on the Work of its Second Session.

